# High-Throughput Sequencing of Human Immunoglobulin Variable Regions with Subtype Identification

**DOI:** 10.1371/journal.pone.0111726

**Published:** 2014-11-03

**Authors:** Merle Schanz, Thomas Liechti, Osvaldo Zagordi, Enkelejda Miho, Sai T. Reddy, Huldrych F. Günthard, Alexandra Trkola, Michael Huber

**Affiliations:** 1 Institute of Medical Virology, University of Zurich, Zurich, Switzerland; 2 Department of Biosystems Science and Engineering, ETH Zurich, Basel, Switzerland; 3 Division of Infectious Diseases and Hospital Epidemiology, University Hospital Zurich, University of Zurich, Zurich, Switzerland; University of Massachusetts Medical Center, United States of America

## Abstract

The humoral immune response plays a critical role in controlling infection, and the rapid adaptation to a broad range of pathogens depends on a highly diverse antibody repertoire. The advent of high-throughput sequencing technologies in the past decade has enabled insights into this immense diversity. However, not only the variable, but also the constant region of antibodies determines their *in vivo* activity. Antibody isotypes differ in effector functions and are thought to play a defining role in elicitation of immune responses, both in natural infection and in vaccination. We have developed an Illumina MiSeq high-throughput sequencing protocol that allows determination of the human IgG subtype alongside sequencing full-length antibody variable heavy chain regions. We thereby took advantage of the Illumina procedure containing two additional short reads as identifiers. By performing paired-end sequencing of the variable regions and customizing one of the identifier sequences to distinguish IgG subtypes, IgG transcripts with linked information of variable regions and IgG subtype can be retrieved. We applied our new method to the analysis of the IgG variable region repertoire from PBMC of an HIV-1 infected individual confirmed to have serum antibody reactivity to the Membrane Proximal External Region (MPER) of gp41. We found that IgG3 subtype frequencies in the memory B cell compartment increased after halted treatment and coincided with increased plasma antibody reactivity against the MPER domain. The sequencing strategy we developed is not restricted to analysis of IgG. It can be adopted for any Ig subtyping and beyond that for any research question where phasing of distant regions on the same amplicon is needed.

## Introduction

In the past decade, the development of high-throughput sequencing technologies (Next Generation Sequencing, NGS) has largely influenced research possibilities in immunology. Sequencing of whole antibody repertoires has become feasible and affordable, offering new approaches to quantitatively study immune responses [1], [2]. For example, the search for potent neutralizing antibodies against human immunodeficiency virus type 1 (HIV-1) and ways to elicit them by vaccination has in recent years funneled extensive research that increasingly relies on NGS of the IgG variable region, which enables high-resolution profiling of antibody repertoires and the evolution of neutralizing antibodies over time [3]–[8].

For immune effector functions, not only the variable part of an antibody is important, but also the different isotypes of the constant region. Antibodies of the same epitope specificity can therefore elicit different effector functions depending on the isotype. Antibody-dependent cell-mediated cytotoxicity (ADCC) for instance is most active with isotype IgG1 followed by IgG3 and IgA. Subtypes of IgG differentially protect mice from bacterial infection [9] and are associated with chikungunya virus clearance and long-term clinical protection [10]. An intriguing example of the potential importance of IgG subtypes for immune reaction and antibody elicitation is the membrane-proximal external region (MPER) of gp41 of HIV-1. All of the broadly neutralizing anti-MPER antibodies identified thus far, 4E10 and 2F5 [11] and the recently identified 10E8 [12], were originally isolated as IgG3. However, in the case of 4E10, the *in vitro* neutralization potency is higher for IgG1 and absent for IgM [13]. It was suggested that this is related to the longer hinge region and greater flexibility of the IgG3 subtype [14], [15]. Of note, in the recent RV144 trial [16], the first phase III trial of an HIV-1 vaccine that reported some efficacy, anti-gp120-specific isotype selection was skewed towards IgG3 [17] and anti-HIV-1 IgG3 antibodies correlated with antiviral function [18]. These examples highlight the importance of evaluating antibody specificity alongside subtype information when studying immune responses and developing vaccines.

The Illumina MiSeq platform is rapidly becoming the dominant sequencing system for antibody repertoires due to low error rates, long read lengths, and declining costs [2]. State of the art sequencing with Illumina technology currently allows for read lengths of 2×300 nucleotides on the widely used MiSeq platform. This is sufficient to sequence an antibody variable region from both ends with an overlap allowing combination of both reads to a full-length variable region. However, the available read length might not be enough for antibodies with a long heavy chain complementary determining region 3 (HCDR3) to also include determinants of the antibody subtype in the sequences, as they are located too far downstream in the constant region. In order to overcome this limitation, we use one of the indexing reads the Illumina technology applies not in its intended function as a sample identifier, but instead as a short extra read that identifies the IgG subtype. This way, we can retrieve full-length variable regions including the IgG subtype. Of note, in the same sequencing runs light chains and other desired heavy chain isotypes can be sequenced. The second Illumina index read is not modified and used as designed to allow analysis of multiple samples in a single run.

## Methods

### Primers

For the heavy chain, forward primers binding to the leader sequences and reverse primers in the constant region were used [6], [19]. For the kappa light chain, primers binding in the leader region [19] and in the constant region were used. Lambda light chains were amplified with primers binding in the leader/variable [19] and in the joining region [20]. Our customized protocol uses sequencing adaptors and index sequences based on the Illumina (San Diego, CA) TruSeq HT setup. Four random nucleotides were inserted between the sequencing adaptor and the specific primer to increase diversity and help cluster identification on the Illumina MiSeq flow cell. The sequences of all primers are listed in Table 1. Primers were ordered HPL-purified from Microsynth AG (Balgach, Switzerland).

**Table 1:** List of PCR and sequencing primers.

IGH forward	Seq5N4-VH1LA	CTTTCCCTACACGACGCTCTTCCGATCTNNNNATGGACTGGACCTGGAGGAT
	Seq5N4-VH1LB	CTTTCCCTACACGACGCTCTTCCGATCTNNNNATGGACTGGACCTGGAGCAT
	Seq5N4-VH1LC	CTTTCCCTACACGACGCTCTTCCGATCTNNNNATGGACTGGACCTGGAGAAT
	Seq5N4-VH1LD	CTTTCCCTACACGACGCTCTTCCGATCTNNNNGGTTCCTCTTTGTGGTGGC
	Seq5N4-VH1LE	CTTTCCCTACACGACGCTCTTCCGATCTNNNNATGGACTGGACCTGGAGGGT
	Seq5N4-VH1LF	CTTTCCCTACACGACGCTCTTCCGATCTNNNNATGGACTGGATTTGGAGGAT
	Seq5N4-VH1LG	CTTTCCCTACACGACGCTCTTCCGATCTNNNNAGGTTCCTCTTTGTGGTGGCAG
	Seq5N4-VH3LA	CTTTCCCTACACGACGCTCTTCCGATCTNNNNTAAAAGGTGTCCAGTGT
	Seq5N4-VH3LB	CTTTCCCTACACGACGCTCTTCCGATCTNNNNTAAGAGGTGTCCAGTGT
	Seq5N4-VH3LC	CTTTCCCTACACGACGCTCTTCCGATCTNNNNTAGAAGGTGTCCAGTGT
	Seq5N4-VH3LD	CTTTCCCTACACGACGCTCTTCCGATCTNNNNGCTATTTTTAAAGGTGTCCAGTGT
	Seq5N4-VH3LE	CTTTCCCTACACGACGCTCTTCCGATCTNNNNTACAAGGTGTCCAGTGT
	Seq5N4-VH3LF	CTTTCCCTACACGACGCTCTTCCGATCTNNNNTTAAAGCTGTCCAGTGT
	Seq5N4-VH4LA	CTTTCCCTACACGACGCTCTTCCGATCTNNNNATGAAACACCTGTGGTTCTTCC
	Seq5N4-VH4LB	CTTTCCCTACACGACGCTCTTCCGATCTNNNNATGAAACACCTGTGGTTCTT
	Seq5N4-VH4LC	CTTTCCCTACACGACGCTCTTCCGATCTNNNNATGAAGCACCTGTGGTTCTT
	Seq5N4-VH4LD	CTTTCCCTACACGACGCTCTTCCGATCTNNNNATGAAACATCTGTGGTTCTT
	Seq5N4-VH5LA	CTTTCCCTACACGACGCTCTTCCGATCTNNNNTTCTCCAAGGAGTCTGT
	Seq5N4-VH5LB	CTTTCCCTACACGACGCTCTTCCGATCTNNNNCCTCCACAGTGAGAGTCTG
	Seq5N4-VH6LA	CTTTCCCTACACGACGCTCTTCCGATCTNNNNATGTCTGTCTCCTTCCTCATC
	Seq5N4-VH7LA	CTTTCCCTACACGACGCTCTTCCGATCTNNNNGGCAGCAGCAACAGGTGCCCA
IGL forward	Seq5N4-VL1	CTTTCCCTACACGACGCTCTTCCGATCTNNNNGGTCCTGGGCCCAGTCTGTGCTG
	Seq5N4-VL2	CTTTCCCTACACGACGCTCTTCCGATCTNNNNGGTCCTGGGCCCAGTCTGCCCTG
	Seq5N4-VL3	CTTTCCCTACACGACGCTCTTCCGATCTNNNNGCTCTGTGACCTCCTATGAGCTG
	Seq5N4-VL45	CTTTCCCTACACGACGCTCTTCCGATCTNNNNGGTCTCTCTCSCAGCyTGTGCTG
	Seq5N4-VL6	CTTTCCCTACACGACGCTCTTCCGATCTNNNNGTTCTTGGGCCAATTTTATGCTG
	Seq5N4-VL7	CTTTCCCTACACGACGCTCTTCCGATCTNNNNGGTCCAATTCyCAGGCTGTGGTG
	Seq5N4-VL8	CTTTCCCTACACGACGCTCTTCCGATCTNNNNGAGTGGATTCTCAGACTGTGGTG
IGK forward	Seq5N4-VK12	CTTTCCCTACACGACGCTCTTCCGATCTNNNNATGAGGSTCCCyGCTCAGCTGCTGG
	Seq5N4-VK3	CTTTCCCTACACGACGCTCTTCCGATCTNNNNCTCTTCCTCCTGCTACTCTGGCTCCCAG
	Seq5N4-VK4	CTTTCCCTACACGACGCTCTTCCGATCTNNNNATTTCTCTGTTGCTCTGGATCTCTG
IGG reverse	TS7IgG(int)	CAAGCAGAAGACGGCATACGAGATccGTTCGGGGAAGTAGTCCTTGAC
IGL reverse	IgGcSeqhuVL1-rev	GGGAAGACCGATGGGCCCTTGGTNNNNTAGGACGGTSASCTTGGTCC
	IgGcSeqhuVL7-rev	GGGAAGACCGATGGGCCCTTGGTNNNNGAGGACGGTCAGCTGGGTGC
IGK reverse	IgGcSeqhuVKC-rev	GGGAAGACCGATGGGCCCTTGGTNNNNAGATGGTGCAGCCACAGTTC
IGM reverse	IgGcSeqhuIgM-rev	GGGAAGACCGATGGGCCCTTGGTNNNNGGTTGGGGCGGATGCACTCC
IGA reverse	IgGcSeqIgA-rev	GGGAAGACCGATGGGCCCTTGGTNNNNTTGGGGCTGGTCGGGGATGC
indexing forward	TS-D501	AATGATACGGCGACCACCGAGATCT ACACTATAGCCTACACTCTTTCCCTA CACGACGCTCTTCCGATCT
	TS-D502	AATGATACGGCGACCACCGAGATCT ACACATAGAGGCACACTCTTTCCCTA CACGACGCTCTTCCGATCT
	TS-D503	AATGATACGGCGACCACCGAGATCT ACACCCTATCCTACACTCTTTCCCTA CACGACGCTCTTCCGATCT
	TS-D504	AATGATACGGCGACCACCGAGATCT ACACGGCTCTGAACACTCTTTCCCTA CACGACGCTCTTCCGATCT
	TS-D505	AATGATACGGCGACCACCGAGATCT ACACAGGCGAAGACACTCTTTCCCTA CACGACGCTCTTCCGATCT
	TS-D506	AATGATACGGCGACCACCGAGATCT ACACTAATCTTAACACTCTTTCCCTA CACGACGCTCTTCCGATCT
	TS-D507	AATGATACGGCGACCACCGAGATCT ACACCAGGACGTACACTCTTTCCCTA CACGACGCTCTTCCGATCT
	TS-D508	AATGATACGGCGACCACCGAGATCT ACACGTACTGACACACTCTTTCCCTA CACGACGCTCTTCCGATCT
klMA indexingreverse	TS7icIgGcSeq	CAAGCAGAAGACGGCATACGAGATT CTCCACGAGAAGGAGGAGGGTGCCA GGGGGAAGACCGATGGGCCCTTGGT
custom sequencing	IgGcSeq	CCAGGGGGAAGACCGAT GGGCCCTTGGT
	IgGcInd	CCATCGGTCTTCCCCCTGGCRCCCTSCTCC

### Clinical specimen

PBMC from healthy donors were purified from buffy coat obtained from the Zurich Blood Transfusion Service (www.zhbsd.ch). Cryopreserved PBMC from an HIV-1 infected individual, patient ZA159, who developed strong MPER specific antibody responses during disease progression (Liechti et al, in preparation), were obtained through the Zurich primary HIV infection (ZPHI) study [21]. BioSample accession numbers for the human subjects are SAMN02911274 to SAMN02911277.

### Ethics statement

Cryopreserved PBMC were obtained from an adult participant enrolled in the Zurich Primary HIV-infection (ZPHI) study (http://clinicaltrials.gov, ID 5 NCT00537966) [21]. The study was approved by the ethics committee of the canton of Zurich and written informed consent was obtained from all participating individuals. Buffy coats from healthy donors were obtained from the Zurich Blood Transfusion Service (www.zhbsd.ch) under a protocol approved by the ethics committee of the canton of Zurich.

### PCR amplification

Total RNA was extracted from 10 * 10^6^ PBMC (healthy donor) or 2 * 10^6^ PBMC (patient) using the RNeasy Mini Kit (Qiagen). cDNA was synthesized in a total volume of 40 ul using 400 U SuperscriptIII, 1 ug Oligo(dT)_15_ primer, 2 ul dNTP mix (10 mM each nucleotide), 2 ul 0.1 M DTT and 1–10 ug RNA. Reverse transcription was performed at 65°C for five minutes, 50°C for 60 minutes and 70°C for 15 minutes. cDNA was stored at −20°C. Ig heavy, kappa and lambda genes were amplified in separate reactions. All PCR reactions were performed in volumes of 50 ul using 0.5 ul Phusion High-Fidelity DNA Polymerase (New England Biolabs), 1 ul dNTPs (10 mM each nucleotide) and 5 ul cDNA template. The first PCR reaction was performed with the forward primer mix (0.05–0.15 uM each primer) and 0.5 uM gene specific reverse primer. The temperature protocol was adapted from [22] and consisted of once 98°C for 60 s; 4 cycles of 98°C 10 s, 45°C 30 s, 72°C 30 s; 4 cycles of 98°C 10 s, 50°C 30 s, 72°C 30 s; 17 cycles of 98°C 10 s, 68°C 30 s, 72°C 30 s; once 72°C 10 min.

The second PCR was performed with the forward index adaptor primers (TS-D501 to TS-D508, depending on the number of indices needed) and two custom reverse primers for either the IgG heavy chains (TS7IgG(int)) or the light chains and heavy chains of other isotypes (TS7icIgGcSeq). The temperature protocol was once 98°C for 60 s; 4 cycles of 98°C 10 s, 55°C 30 s, 72°C 30 s; 4 cycles of 98°C 10 s, 60°C 30 s, 72°C 30 s; 17 cycles of 98°C 10 s, 72°C 30 s; once 72°C 10 min. The four different healthy donor preparations differed in their amplification strategies: prep 4 was amplified as described above, preps 1 and 2 were amplified with 1 ul cDNA template, prep 3 with 5 ul cDNA template. Preps 1, 2 and 3 were then amplified for 12, 25 and 12 cycles in the second PCR, respectively. Samples from sorted cells were amplified for 40 cycles in the first PCR.

The amplicons were purified using the QIAquick Gel Extraction Kit (Qiagen). Samples were quantitated using Quant-iT PicoGreen (Invitrogen, Carlsbad, CA), normalized to a concentration of 4 nM (based on an average length of about 525 nucleotides for light chains and 595 nucleotides for heavy chains) and pooled equimolar for sequencing.

### Sequencing strategy

**Figure 1:**
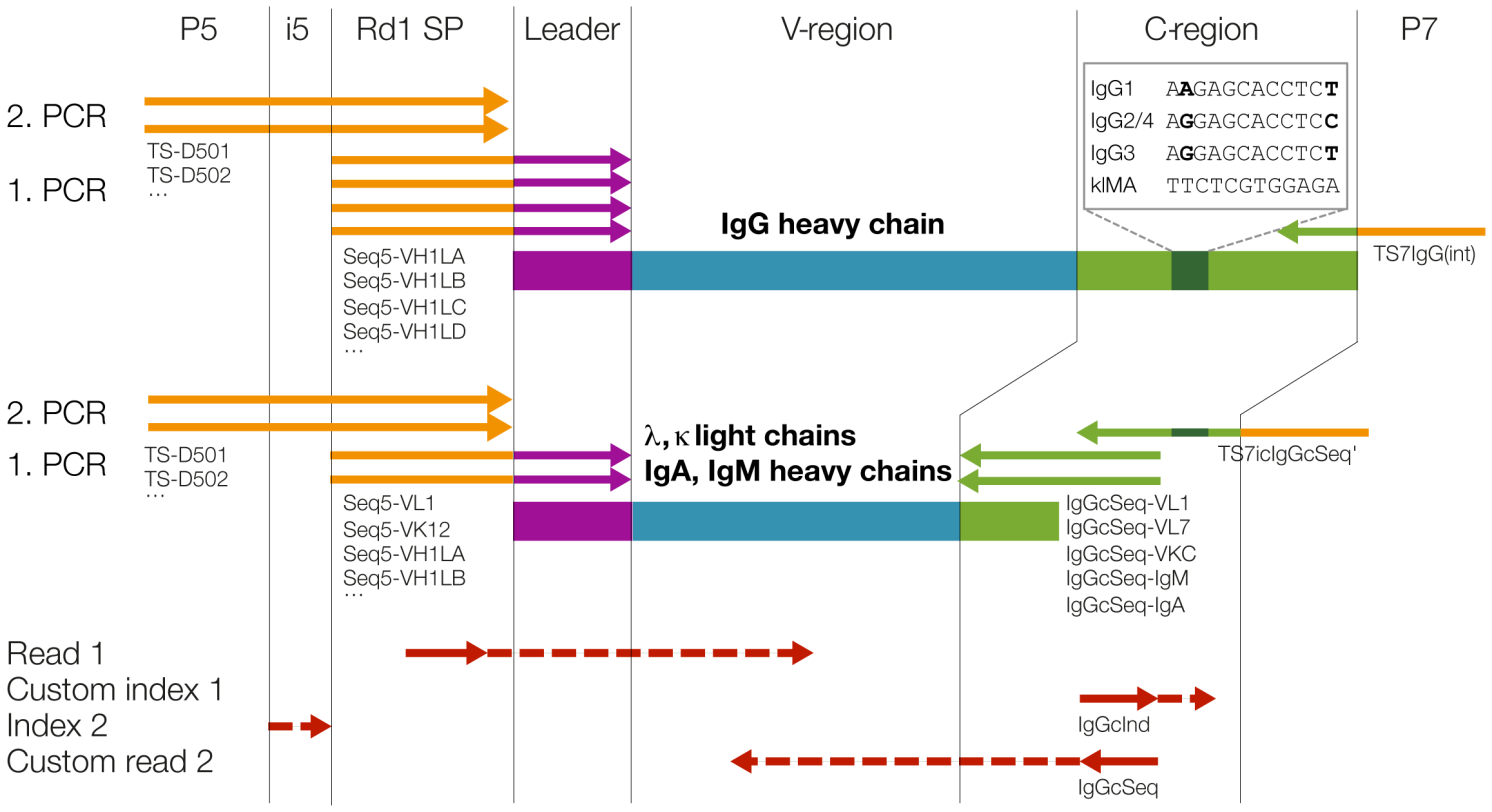
Amplification of Ig variable regions and high-throughput sequencing with subtype information. Antibody heavy and light chain genes are shown schematically with leader regions in purple, variable (V-) regions in blue and constant (C-) regions in green. Ig heavy, kappa and lambda light chain genes were amplified in separate reactions with family specific primers (represented by arrows) binding to leader and constant region. Primer names are indicated exemplarily below the arrows and a complete list of all primers used can be found in Table 1. Sequencing adaptors essential for the Illumina platform (flow cell binding sites P5 and P7, the index 2 region i5 and the read 1 sequencing primer binding site Rd1 SP, illustrated in orange) were added by primer extension during a second PCR reaction, except for the IgG reverse primer TS7IgG(int) which contained an adaptor and was used for both amplifications. Purified libraries were then sequenced using standard Illumina MiSeq primers for read 1 and index 2, and customized primers for index 1 and read 2 (sequencing primers are shown in red and regions sequenced with red dashed arrows).

In Illumina high-throughput sequencing technology, the DNA insert to be sequenced is flanked on both sides by a primer binding site, a short index and an adapter for binding to the flow cell. Conventional use allows for paired-end sequencing (forward read 1 and reverse read 2) and dual multiplexing (index read 1 and index read 2) by four independent sequencing reactions. On the MiSeq system, custom primers for read 1, read 2 and for the index read 1 can be used optionally. The priming of index read 2 cannot be customized. Further, the number of cycles can be individually chosen for all four reads, as long as the sum is not more than 25 cycles higher than the capacity of the kit used (available kits range from 50–600 cycles). We used these features of the MiSeq system to sequence the variable region of immunoglobulins in a paired-end fashion, determined the subtype of IgGs via a 12 nucleotide long identifier read and multiplexed samples by an 8 nucleotide long index read (Figure 1).

**Figure 2:**
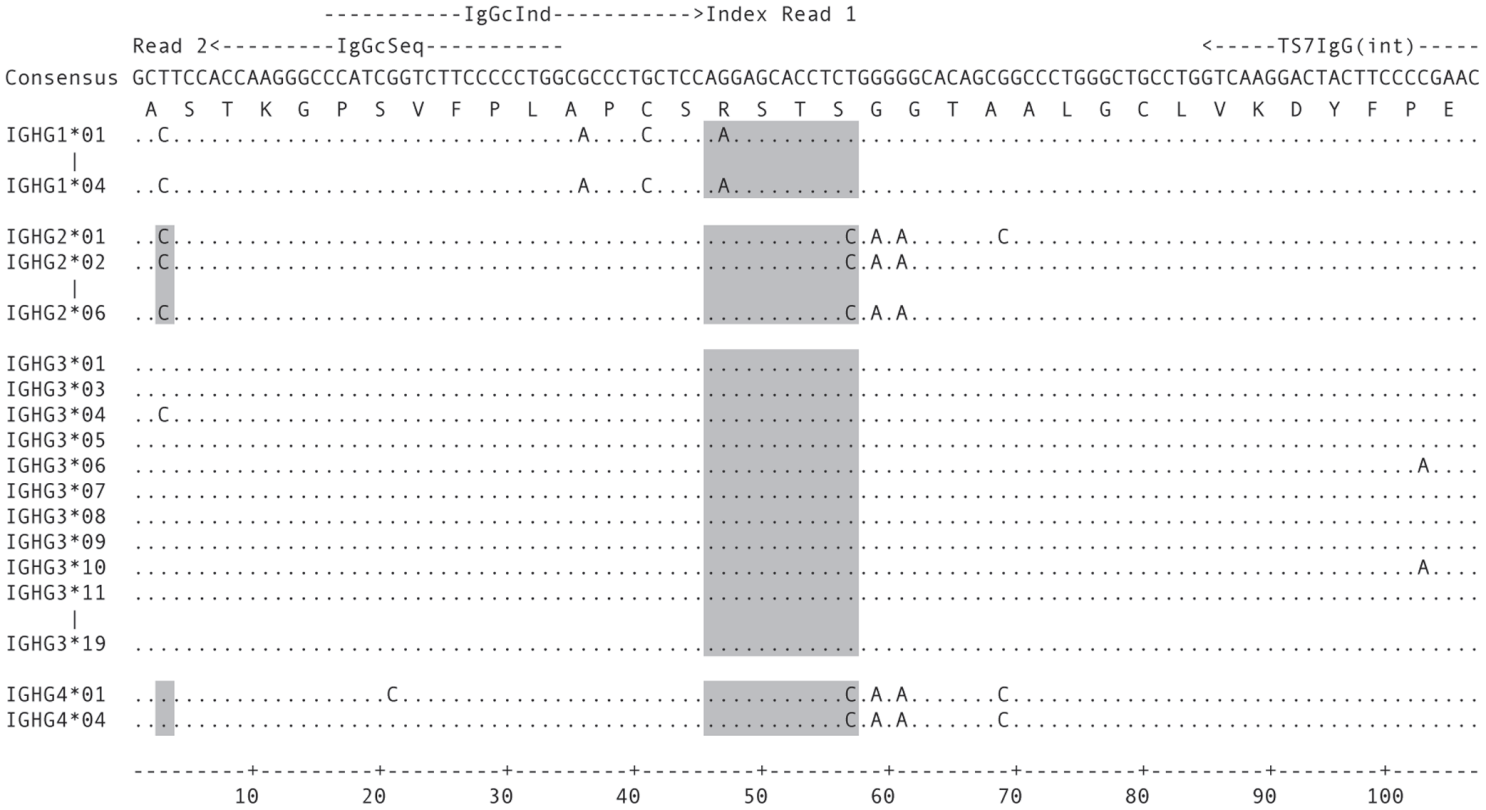
Determinants of IgG subtype in the constant region. IgG1 to IgG4 CH1 regions were obtained from IMGT/GENE-DB [34]. Only the first 107 nucleotides of the constant region are shown. Some alleles identical in this section have been omitted. Binding sites of the IgG reverse primer (TS7IgG(int)) used for library preparation and the custom sequencing and indexing primers (IgGcSeq and IgGcInd, respectively) are represented by dashed arrows on top. The full index 1 and the start of read 2 are indicated. Regions used for subtype assignment are shaded.

The constant region of subtype IgG1 differs from IgG2/3/4 at position 47 (AAG (K) vs. AGG (R), Figure 2). IgG1/3 and IgG2/4 differ at position 57 (TCT (S) vs. TCC (S)). By sequencing this stretch of the constant region and defining the corresponding sequences as indices, subtypes IgG1, 2/4 and 3 can be differentiated. It is not possible to distinguish IgG2 and 4 at this position; however, they can be separated based on the first triplet of the constant region (GCC (A) vs. GCT (A), Figure 2). This way, all four IgG subtypes can be called unequivocally. To make sequencing of light chains and heavy chain isotypes IgM and IgA possible in the same run, the 5′ end of the IgG constant region (nucleotides 7–45) was added into the respective reverse primers so that the same read 2 custom primer could be used for sequencing of IgG, IgA, IgM and kappa (k) and lambda (l) light chains. A separate index (klMA), which is complementary to the IgG1 index to increase base variability during sequencing, was used for those chains.

### Illumina MiSeq sequencing

Pooled samples were denatured with NaOH according to the protocol (Illumina), diluted with hybridization buffer HT1 to a final dilution of 10 pM, spiked with 5% of PhiX control library and loaded into a 500 cycle version 2 reagent cartridge. Custom primers IgGcSeq and IgGcInd for the read 2 and the indexing read, respectively, were diluted to 0.5 uM in hybridization buffer HT1 and 600 ul loaded into well 19 (index read 1, IgGcInd) and well 20 (read 2, IgGcSeq) of the reagent cartridge. The sample sheet was adapted manually to allow any sequence (N_12_) as custom index 1. Sequencing was performed for 2 * 250 cycles. The workflow was set to “GenerateFASTQ”. The raw sequencing data have been uploaded to zenodo (doi:10.5281/zenodo.10863).

### Data analysis

In order to obtain fastq files also for the index reads, “CreateFastqForIndexReads” in the MiSeqReporter.exe.config file was set to 1 (true). Reads were first de-multiplexed by Illumina MiSeq Reporter (version 2.4.60) based on index 2 that distinguishes the different samples. Secondly, reads were assigned to the different subtypes using a python script (available here https://gist.github.com/ozagordi/11180835) as follows: IgG1, IgG3 and light chains or heavy chains of other isotypes (klMA) were identified by their index 1; IgG2 and IgG4 were additionally discriminated based on the fourth nucleotide of the second read (IgG2 if ‘G’, IgG4 if ‘A’, read 2 is reverse complementary). For the IgG subtype indices a perfect match was required, for the klMA index one mismatch was allowed. Reads not matching above criteria were classified as undetermined. Forward and reverse reads of a corresponding pair were stitched together using PANDAseq [23] with a minimal overlap of 10 nucleotides and analyzed by IMGT/HighV-QUEST [24]. Subtype frequencies were calculated as the percentage of completely indexed and full-length Ig variable region rearrangements.

### Staining and cell sorting

Healthy donor PBMC were thawed, washed and split into four samples. Staining for IgG subtypes was performed in PBS/1% FCS at 4°C in the dark for 15 minutes using the following antibodies and dyes: anti-CD19 V500 (BD Horizon), anti-CD3 APC-Cy7 (BioLegend), anti-CD14 APC-Cy7 (BioLegend), LIVE/DEAD Near-IR Dead Cell Stain (Molecular Probes), anti-CD16 APC-Cy7 (BioLegend), anti-IgD PE-Cy5 (BioLegend, labeled in-house) and either anti-IgG1 PE, anti-IgG2 PE, anti-IgG3 PE or anti-IgG4 PE (all from SouthernBiotech). Cells were washed twice, re-suspended in PBS/1% FCS and cells gated for CD3/14/16/Dead^-^ CD19^+^ IgD^-^ and positive for one of the IgG subtypes were sorted on a FACSAriaIII (Becton Dickinson). Sorted cells were frozen at −80°C as dry pellets prior to analysis.

## Results

### Validation of high-throughput immunoglobulin variable region sequencing with subtype identification

We developed a high-throughput method for the Illumina MiSeq system to sequence the full variable region of immunoglobulins in a paired-end fashion and identify at the same time the subtype of IgGs via a 12 nucleotide long custom index read (Figure 1). In order to test our sequencing strategy, we sequenced IgG heavy and light chains from PBMC from a healthy donor and an HIV-1 infected individual (ZA159 week 213, see below). The healthy donor sample was amplified in four separate reactions using different PCR conditions and cDNA input (see methods) to confirm the robustness of the IgG subtype assignment. We focused on assigning IgG subtypes and therefore did not sequence light chains for preparations 1–3. Sequencing of the five samples yielded a total of 10'249'237 passing filter reads.

19.3% (1'981'155) of the paired-end reads could not be demultiplexed to one of the five samples and were categorized as undetermined in regard of sample (Table 2). However, most of these undetermined reads (1′381′101, 13.5% of total reads) had an index identical to the TruSeq Universal primer and were confirmed to be mostly PhiX control reads (data not shown). The high number of undetermined reads therefore results from high PhiX concentrations and not from problems associated with sample preparation or library generation.

**Table 2:** Read numbers and subtype frequencies.

Sample	Subtype	Subtype assignedread pairs	Sequencesafter PANDAseq	Rearrangedvariable regions	IgG subtypesper sample
Healthy donor prep 1	IgG1	529'390	515'758	505'942	56.3%
	IgG2	369'741	360'472	354'751	39.5%
	IgG3	36'604	35'620	34'920	3.9%
	IgG4	3'785	3'645	3'572	0.4%
	klMA	nd	nd	nd	na
	Undet(b)	11'577	na	na	na
Healthy donor prep 2	IgG1	692'673	674'590	660'899	55.9%
	IgG2	488'483	475'851	467'958	39.6%
	IgG3	50'454	48'880	47'773	4.0%
	IgG4	6'191	5'968	5'859	0.5%
	klMA	nd	nd	nd	na
	Undet(b)	18'326	na	na	na
Healthy donor prep 3	IgG1	641'364	623'522	611'013	55.9%
	IgG2	453'471	441'108	433'565	39.7%
	IgG3	46'361	44'911	43'848	4.0%
	IgG4	5'386	5'155	5'030	0.5%
	klMA	nd	nd	nd	na
	Undet(b)	18'149	na	na	na
Healthy donor prep 4	IgG1	699'378	679'819	665'267	56.3%
	IgG2	481'592	468'433	460'214	39.0%
	IgG3	52'028	50'328	49'024	4.2%
	IgG4	7'199	6'900	6'695	0.6%
	klMA	1'317'918	1'257'301	1'210'698	na
	Undet(b)	84'941	na	na	na
ZA159 (week 213)	IgG1	677'787	659'884	646'977	65.8%
	IgG2	183'718	178'800	175'718	17.9%
	IgG3	163'839	158'878	155'704	15.8%
	IgG4	4'487	4'366	4'211	0.4%
	klMA	1'151'587	1'097'762	1'053'604	na
	Undet(b)	71'653	na	na	na
Undet (a)		1'981'155	na	na	na
Total reads		10'249'237	7'797'951	7'603'242	

a) Undetermined in regard of sample.

b) Undetermined in regard of subtype.

nd = not done.

na = not applicable.

IgG subtype assignment based on index read 1 and the first triplet of the constant region sequenced in read 2 resulted in 6 categories (IgG1, IgG2, IgG3, IgG4, klMA, undetermined reads) for each sample (Table 2, column “Subtype assigned read pairs”). Of all read pairs demultiplexed to a sample, 97.5% (8'063'436) were successfully assigned to one of the IgG subtypes or the light chains.

To assemble full-length variable region sequences, corresponding paired end reads were combined with PANDAseq [23]. The overlap of reads peaked at about 100 nucleotides for heavy chains and at about 100 and 150 nucleotides for kappa and lambda light chains, respectively. 96.7% of all the read pairs overlapped (Table 2, column “Sequences after PANDAseq”). Sequences were subjected to IMGT analysis. On average, 98% of both heavy and light chain sequences could be assigned to antibody variable regions. The median heavy chain variable region length in our dataset was approximately 360 nucleotides. In total, 7′603′242 subtype-assigned variable region sequences were obtained (Table 2, column “Rearranged variable regions”), showing that our strategy efficiently sequences full-length variable regions with linked subtype information.

**Figure 3:**
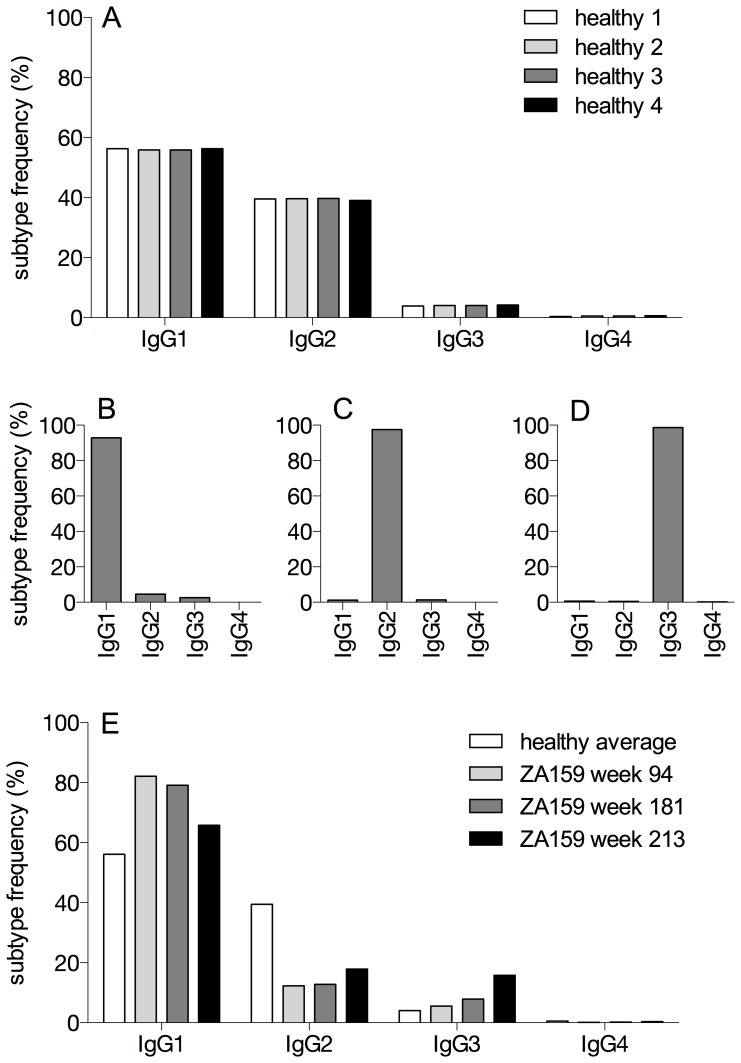
IgG subtypes are reliably identified. A) IgG subtype frequencies of four preparations of PBMC from one healthy donor were determined by sequencing. Different PCR protocols (prep 1 to prep 4) as described in the methods section have been applied to amplify antibody transcripts. Read numbers for the different preparations are listed in Table 2. B–D) PBMC of a healthy control were sorted by FACS into individual IgG subtype populations and sequenced. 2′221′006, 2′044′153 and 1′889′353 reads were obtained for sorted IgG1 positive cells (B), IgG2 positive cells (C) and IgG3 positive cells (D), respectively, and assigned to the IgG subtypes. E) IgG subtype frequencies of time points week 94, week 181 and week 213 (Table 2, Table S1) for patient ZA159. The average frequencies from the healthy control preparations 1–4 are shown as a comparison. Subtype frequencies in all panels were calculated as the percentage of all completely indexed and full-length variable region rearrangements.

The IgG subtype frequencies were found to be very consistent among the four preparations of healthy donor and therefore independent of PCR amplification strategies and cDNA input (Table 2, Figure 3A, average frequency ± std. deviation (%) for IgG1 equals 56.1±0.2, IgG2 39.5±0.3, IgG3 4.0±0.1, IgG4 0.5±0.1). These values correspond well to IgG subtype frequencies previously reported [25]–[27].

### Validation of Ig subtype distribution analysis by NGS and FACS sorting

To confirm the correct calling of the IgG subtype, PBMC of a healthy donor were FACS sorted into the four different IgG subtype populations. Purity after sorting was >99% for CD19^+^ IgG1, IgG2 and IgG3 positive cells (approximately 17000, 7000 and 4500 cells were sorted, respectively). IgG4 positive cells were not further analyzed due to the low yield (total of 82 cells sorted) and lack of possibility to assess post-sort purity by FACS. After high-throughput sequencing of these populations in a separated run and analysis by the same pipeline as described above, we found subtype frequencies of 92.8% IgG1, 97.5% IgG2 and 98.7% IgG3 for the IgG1, IgG2 and IgG3 sorted populations, respectively, highlighting the high specificity of our sequencing strategy (Figure 3 BCD).

### IgG subtype dynamics in an HIV-1 infected patient

To get an insight if our method is applicable to monitor IgG subtype dynamics during infections, we selected an HIV-1 infected patient with pronounced IgG3-mediated anti-MPER plasma antibody response (Liechti et al. in preparation). Patient ZA159 was enrolled in the Zurich primary HIV infection study and has been followed from the acute phase of HIV-1 infection onwards [21]. The patient was on anti-retroviral treatment until week 92 post infection. Samples for NGS analysis were selected from three time points with differential IgG3-mediated MPER plasma titers: the first sample was taken 94 weeks post infection where no IgG3 MPER reactivity was apparent. Plasma from the second time point, approximately 181 weeks post infection, showed intermediate IgG3 MPER reactivity and the third, approximately 213 weeks post infection, had highest IgG3 MPER reactivity.

In addition to the wk213 sample already sequenced in the first run, frozen PBMC from the other two time points were sequenced in a second run and 732'390 and 669'244 heavy chain reads were obtained for those samples from week 94 and 181, respectively (see Table S1 for reads statistics). Assigning these reads to the IgG subtypes and comparing subtype frequencies to those from the healthy donor showed higher IgG1 and decreased IgG2 frequencies for the HIV-1 infected individual at all time points measured (Figure 3A). Of note, during viral rebound after anti-retroviral treatment cessation, IgG3 frequencies (measured by NGS) in the memory B cell compartment increased markedly (Figure 3E), which coincided with the increase in plasma IgG3 MPER reactivity.

## Discussion

Repertoire analysis of antibody variable genes by NGS has become an important tool that allows unprecedented insight into antibody development pathways and holds particular promise for tailored vaccine design. Here, we describe a strategy for high-throughput sequencing of antibody heavy chains including determination of the IgG subtype. To achieve this, we adapted the Illumina MiSeq standard protocol by employing a mixed strategy of index reads and customized primers. Sequencing with custom primers and indices has been done previously [28], [29], but to our knowledge our strategy of using an index read as a “third read” is novel. Different samples can still be multiplexed in the same run as the second index read remains available.

As we demonstrate here, our method determines IgG subtypes very reliably. We could successfully assign 97.5% of the reads in the demultiplexed samples to a subtype, although our identifier is 12 nucleotides long and the assignment criteria have to be very strict. Consistently, over 96% of both heavy and light chain sequences could be assigned to rearranged variable regions, demonstrating that our sequences are full-length antibody variable regions.

As the Hamming distances between the subtype identifiers are only single nucleotides, we do not allow mismatches in the subtype indices, except one mismatch in calling the klMA category. There remains a risk of misidentification of the subtype by a PCR or sequencing error, artifact recombination [30] or a mutation in the constant region. If further exclusion of misidentification by sequencing errors in the indices should become warranted for specific research questions, our analysis could be adapted to first collapse identical variable regions and then use the consensus of their index reads to determine the subtype. While this approach would decrease potential misidentifications, a full repertoire analysis of the variable domains would be required. Although this was beyond the scope of our current study, we consider this a useful and valuable modification of our analysis for future projects. Yet, despite the increased accuracy of this approach, pre-existing mutations in the constant region will not be detected and dismissed. Another possibility to empower subtype identification would be full-length sequencing of the CH1, as the difference between subtypes over the whole CH1 would increase to 6–15 nucleotides. However, the required read lengths are currently limiting for Illumina technology, as additional sequencing of the CH1 domain further downstream of our index would be necessary [27]. Even if this became possible, splitting up the available read length in several smaller reads might still be preferable, as per base sequencing quality decreases with increasing read length.

Although the purity of sorted subtype populations was higher measured by FACS than the IgG subtype frequency in the sorted samples determined by our sequencing approach, we argue that the sequencing approach serves as a quality control for the sorting and not the other way round, as even in the most controlled set up, FACS sorting will suffer from residual cross-reactivity from the staining antibodies.

No bias or cross-reactivity is expected in sequencing as this method is independent of immunoglobulin surface expression and, importantly, all subtypes are amplified with the same primer. A common primer is a key advantage compared to individual primers for each subtype. It is, however, important to note that our method, as it is presented here, is only semi-quantitative as we focused solely on subclass determination. If needed, a quantitative analysis would require a full repertoire analysis to avoid counting the same variable region multiple times. Since oversampling should be proportional for all the subtypes, distribution of subtype frequencies as shown here should not be affected.

Our method has the potential of widespread application and particularly in the antibody field the chance to fill a gap in information. So far, antibody subtypes have either only been determined in bulk in plasma samples where the information could not be linked to variable region sequences, or on the level of antibodies cloned out of single cells, where the potential for high-throughput applications is limited.

As recent data have highlighted, information on IgG subtype profiles could be very useful to study elicitation and dynamics of IgG antibodies of different subtypes, and could provide information on the quality of infection- and vaccine-induced B cell responses [18], [31], [32].

Our method can easily be adapted for IgA subtype discrimination. It can also be applied in other cases where priming of three reads is necessary or sequence information of a distant site is needed, e.g. in haplotype analysis used in genetics. Overall, our method combines the strength of antibody repertoire analyses by NGS with subtype information of the obtained sequences, enabling in-depth analysis of immune responses following infections or vaccinations.

## Supporting Information

Table S1 **Read numbers and subtype frequencies (ZA159 week 94 and 181).**
(DOCX)
