# Supplementary material for: High-Throughput Sequencing of Human Immunoglobulin Variable Regions with Subtype Identification
Source: PLoS One. 2014 Nov 3;9(11):e111726. doi: 10.1371/journal.pone.0111726 (PMC4218849; doi:10.1371/journal.pone.0111726)
Supplement: Table S1 — Read numbers and subtype frequencies (ZA159 week 94 and 181). (DOCX) [file pone.0111726.s001.docx]

**Table S1: Read numbers and subtype frequencies (ZA159 week 94 and 181)**

| Sample | Subtype | Subtype assigned read pairs | Sequences after PANDAseq | Rearranged variable regions | IgG subtypes per sample |
| --- | --- | --- | --- | --- | --- |
| ZA159 week 94 | IgG1 | 641'130 | 625'783 | 622'364 | 82.1% |
|  | IgG2 | 95'243 | 93'488 | 93'344 | 12.3% |
|  | IgG3 | 42'767 | 42'087 | 41'878 | 5.5% |
|  | IgG4 | 840 | 822 | 804 | 0.1% |
|  | klMA | 296'751 | 258'461 | 250'684 | na |
|  | Undet (a) | 38'741 | na | na | na |
| ZA159 week 181 | IgG1 | 554'162 | 539'583 | 536'244 | 79.1% |
|  | IgG2 | 89'257 | 86'896 | 86'622 | 12.8% |
|  | IgG3 | 56'165 | 54'120 | 53'475 | 7.9% |
|  | IgG4 | 1'390 | 1'318 | 1'260 | 0.2% |
|  | klMA | 285'235 | 252'933 | 243'661 | na |
|  | Undet (a) | 35'593 | na | na | na |

a) Undetermined in regard of subtype

na = not applicable
